# Bacterial Composition and Diversity in Breast Milk Samples from Mothers Living in Taiwan and Mainland China

**DOI:** 10.3389/fmicb.2017.00965

**Published:** 2017-05-30

**Authors:** Shiao-Wen Li, Koichi Watanabe, Chih-Chieh Hsu, Shiou-Huei Chao, Zheng-Hua Yang, Yan-Jun Lin, Chun-Chiang Chen, Yong-Mei Cao, Hsuan-Cheng Huang, Chuan-Hsiung Chang, Ying-Chieh Tsai

**Affiliations:** ^1^Institute of Biomedical Informatics, National Yang-Ming UniversityTaipei, Taiwan; ^2^Bioinformatics Program, Taiwan International Graduate Program, Institute of Information Science, Academia SinicaTaipei, Taiwan; ^3^Department of Animal Science and Technology, National Taiwan UniversityTaipei, Taiwan; ^4^Bioresource Collection and Research Center, Food Industry Research and Development InstituteHsinchu, Taiwan; ^5^Bened Biomedical Co. LtdTaipei, Taiwan; ^6^Institute of Biochemistry and Molecular Biology, National Yang-Ming UniversityTaipei, Taiwan; ^7^Research and Development, Want Want China Holdings LtdShanghai, China

**Keywords:** 16S rRNA gene sequencing, human breast milk, microbiota, Taiwan, Mainland China, vaginal delivery, cesarean section

## Abstract

Human breast milk is widely recognized as the best source of nutrients for healthy growth and development of infants; it contains a diverse microbiota. Here, we characterized the diversity of the microbiota in the breast milk of East Asian women and assessed whether delivery mode influenced the microbiota in the milk of healthy breast-feeding mothers. We profiled the microbiota in breast milk samples collected from 133 healthy mothers in Taiwan and in six regions of mainland China (Central, East, North, Northeast, South, and Southwest China) by using 16S rRNA pyrosequencing. Lactation stage (months postpartum when the milk sample was collected) and maternal body mass index did not influence the breast milk microbiota. Bacterial composition at the family level differed significantly among samples from the seven geographical regions. The five most predominant bacterial families were *Streptococcaceae* (mean relative abundance: 24.4%), *Pseudomonadaceae* (14.0%), *Staphylococcaceae* (12.2%), *Lactobacillaceae* (6.2%), and *Oxalobacteraceae* (4.8%). The microbial profiles were classified into three clusters, driven by *Staphylococcaceae* (abundance in Cluster 1: 42.1%), *Streptococcaceae* (Cluster 2: 48.5%), or *Pseudomonadaceae* (Cluster 3: 26.5%). Microbial network analysis at the genus level revealed that the abundances of the Gram-positive *Staphylococcus*, *Streptococcus*, and *Rothia* were negatively correlated with those of the Gram-negative *Acinetobacter*, *Bacteroides*, *Halomonas*, *Herbaspirillum*, and *Pseudomonas*. Milk from mothers who had undergone Caesarian section (C-section group) had a significantly higher abundance of *Lactobacillus* (*P* < 0.05) and a higher number of unique unclassified operational taxonomic units (OTUs) (*P* < 0.001) than that from mothers who had undergone vaginal delivery (vaginal group). These findings revealed that (i) geographic differences in the microbial profiles were found in breast milk from mothers living in Taiwan and mainland China, (ii) the predominant bacterial families *Streptococcaceae*, *Staphylococcaceae*, and *Pseudomonadaceae* were key components for forming three respective clusters, and (iii) a significantly greater number of unique OTUs was found in the breast milk from mothers who had undergone C-section than from those who had delivered vaginally.

## Introduction

Human breast milk is recognized as the best source of nutrients for healthy growth and development of infants because it contains bioactive components, including oligosaccharides (Bode, 2012; Ballard and Morrow, 2013). Breastfeeding protects against gastrointestinal infections (Ladomenou et al., 2010; Schnitzer et al., 2014) and is associated with a lower incidence of necrotizing enterocolitis (Meinzen-Derr et al., 2009; Herrmann and Carroll, 2014), respiratory infections (Duijts et al., 2010), and allergic diseases (Jeurink et al., 2012; Munblit and Verhasselt, 2016). Several studies have shown that human breast milk contains commensal bacteria that may have beneficial effects on the newborn infant (Heikkilä and Saris, 2003; Gueimonde et al., 2007; Martín et al., 2007a,b, 2009; Perez et al., 2007; Collado et al., 2009; Hunt et al., 2011).

Milk from healthy women contains 10^3^–10^5^ cfu/ml viable bacteria, as revealed by using culture-dependent methods (Martín et al., 2003; Perez et al., 2007). Therefore, breastfeeding is a continuous source of potentially beneficial microbiota. Lactobacilli (10^3^–10^4^ cells/ml) and bifidobacteria (10^2^–10^5^ cells/ml) have been detected by quantitative polymerase chain reaction (qPCR) in human milk (Gueimonde et al., 2007; Martín et al., 2009; Collado et al., 2012; Tuzun et al., 2013; Khodayar-Pardo et al., 2014; Cabrera-Rubio et al., 2016). Recently, next-generation sequencing (NGS) has been adopted for microbial identification by profiling of the 16S rRNA gene; NGS is a more sensitive and less biased analytical method than qPCR, and has been used to analyze the diversity and temporal stability of the bacterial community in human milk (Hunt et al., 2011; Jost et al., 2013; Ward et al., 2013; Jiménez et al., 2015). *Streptococcus*, *Staphylococcus*, and *Propionibacterium* have been confirmed as the “core genera” in breast milk (Hunt et al., 2011; Jiménez et al., 2015).

It is well known that differences in geographic locations and in the times and methods of milk collection affect the results of analysis of breast milk microbiota. Kumar et al. (2016) found apparent geographical differences between countries (China, Finland, South Africa, and Spain) in the fatty acid and microbial compositions of breast milk samples. Changes in the breast milk microbiota are also associated with mode of delivery, and significant differences were found in milk microbiota profiles between the two delivery mode groups (vaginal delivery vs. Cesarean section) (Cabrera-Rubio et al., 2012, 2016; Kumar et al., 2016).

These studies have shown that the bacterial diversity in human milk is higher than was previously assumed; a common conclusion has been that a “core” set of microbiota may exist in human milk, as in other bacterial communities in the human body (Turnbaugh et al., 2009; Ravel et al., 2011). Fitzstevens et al. (2016) suggested that two genera, *Streptococcus* and *Staphylococcus*, may be universally predominant in human milk, regardless of differences in geographic location or analytical methods. However, in fact, varied results for the whole microbial composition have been obtained from previous studies performed in different countries by using different subjects and different analytical methods.

Here, to consolidate these findings and elucidate in detail the features of breast milk microbiota that are potentially important determinants of infant development, we aimed to: (i) characterize the diversity of the microbiota in the breast milk of East Asian women; and (ii) assess whether delivery mode influences the microbiota in the milk of healthy breast-feeding mothers. We profiled the microbiota in breast milk samples collected from healthy mothers in Taiwan and mainland China by using 16S rRNA gene pyrosequencing.

## Materials and Methods

### Sample Collection and Processing

A total of 133 mothers living in Taiwan and six regions of mainland China, who had delivered within a year, provided milk samples (Supplementary Table S1). This study was conducted according to the guidelines of the Declaration of Helsinki. All of the procedures involving human subjects were approved by the ethics committees of Fudan University, China (Reference number: 2013101) and Taipei Veterans General Hospital, Taiwan (Reference number: 2012-08-010AY). Written informed consent was obtained from all participants who provided the milk samples used. We entered and analyzed all samples and questionnaire data anonymously and will publish all data anonymously by using personal numbers.

All participants were non-smokers and had no infectious diseases requiring medical attention in the 2 weeks before milk collection. Milk samples were collected according to a standard protocol and were promptly transported to the laboratory under refrigerated conditions. Briefly, before sample collection, wearing sterile gloves, the women cleaned the nipple and surrounding area with an alcohol swab to minimize the presence of skin bacteria. Milk was collected into a sterile tube by using a handheld electric breast pump (SCF312/01, Philips Avent, Amsterdam, The Netherlands). The first few drops of manually expressed milk were discarded. The part of the pump that contacted the breast, and all of the materials and the device used to collect the milk, were cleansed well and sterilized before each use. Sampling was performed twice, with 75 ml collected each time from both nipples (150 ml from each mother in total). Milk samples (6 ml) were mixed with 6 ml RNA stabilization solution (RNA*later*; Ambion, Inc., Austin, TX, United States) for DNA extraction. Samples were stored in sterile plastic tubes at 4°C until delivery to the laboratory (within 1 h after collection) and then at -70°C until DNA extraction.

### DNA Extraction

Bacterial DNA was extracted from samples by using the bead-beating method and purified as described previously (Nakayama et al., 2015), with slight modification. Two milliliters of thawed sample was centrifuged at 20,000 ×*g* for 5 min at 4°C, and the pellet was resuspended in a solution containing 450 μl of extraction buffer (200 mM Tris-HCl, 80 mM EDTA; pH 9.0) and 50 μl of 10% sodium dodecyl sulfate. Glass beads (300 mg; diameter, 0.1 mm) (Tomy Seiko Co., Ltd, Tokyo, Japan) and 500 μl of TE buffer–saturated phenol were added to the suspension, and the mixture was vortexed vigorously for 30 s by using a FastPrep 24 Instrument (MP Biomedicals, Irvine, CA, United States) at a speed of 5.0 m/s. A phenol – chloroform – isoamyl alcohol mixture (400 μl; 25:24:1; v/v) was added to 400 μl of the supernatant and the sample was shaken vigorously by the FastPrep 24 at 4.0 m/s for 45 s. Samples were centrifuged at 20,000 ×*g* for 5 min at 4°C. Each supernatant (250 μl) was mixed with 25 μl of 3 M sodium acetate (pH 5.2) and kept for 3 min on ice; ice-cold 100% isopropanol (300 μl) was then added. The samples were centrifuged as above. Each DNA pellet was washed in 500 μl of ice-cold 70% ethanol, air-dried, and resuspended in TE buffer (pH 8.0). The DNA concentration was adjusted to 10 μg/ml and the DNA was stored at -20°C.

### Pyrotag Sequencing of 16S rRNA Genes

The PCR conditions were designed as previously described (Oki et al., 2014) with slight modification. The V1–V2 region of the bacterial 16S rRNA gene was amplified by PCR with a bacterial universal primer set (27F-mod: 5′-AGRGTTTGATYMTGGCTCAG-3′ and 338R: 5′-TGCTGCCTCCCGTAGGAGT-3′) (Kim et al., 2013). For the first PCR step, the reaction mixture (25 μl) contained DNA (10 ng), 10 mM Tris-HCl (pH 8.3), 50 mM KCl, 2 mM MgCl_2_, 200 μM of each deoxynucleoside triphosphate (dNTP), 5 pmol of each primer, and 0.625 U Ex *Taq* HS (Takara Bio, Shiga, Japan). The PCR conditions were as follows: 98°C for 2.5 min; 15 cycles of 98°C for 15 s, 50°C for 30 s, and 72°C for 20 s; and finally 72°C for 5 min. For the second PCR, 27Fmod with 128 different 10-bp barcode sequence tags (Nakayama, 2010) and 338R were used. Each primer had an additional adapter sequence at its 5′-end; this was required for the subsequent pyrosequencing reactions. The PCR mixture (50 μl) contained DNA (10–100 ng), 10 mM Tris-HCl (pH 8.3), 50 mM KCl, 1.5 mM MgCl_2_, 200 μM of each dNTP, 10 pmol of each primer, and 1.25 U TaKaRa Ex *Taq* HS (Takara Bio). The PCR conditions were as follows: 98°C for 2.5 min; 20 cycles at 98°C for 15 s, 54°C for 30 s, and 72°C for 20 s; and finally 72°C for 5 min. The PCR products were purified by using a QIAquick PCR Purification Kit (Qiagen, Valencia, CA, United States) according to the manufacturer’s protocol. The purified products were quantified by using a NanoDrop ND-1000 spectrophotometer (NanoDrop Technologies, Wilmington, DE, United States). Amplicons from different samples (100 ng each) were pooled, purified by standard ethanol precipitation, and amplified by emulsion PCR with a GS FLX Titanium LV emPCR Kit (Lib-L) v2 according to the manufacturer’s protocol (454 Life Sciences/Roche Diagnostics, Basel, Switzerland). Sequencing was performed with an FLX Genome Sequencer (454 Life Sciences) with a GS FLX Titanium Sequencing Kit XLR70 according to the manufacturer’s protocol (454 Life Sciences).

### Pyrotag Sequencing Data Processing

16S rRNA gene sequences were processed by using the Quantitative Insights Into Microbial Ecology (QIIME v1.9.0) pipeline (Caporaso et al., 2010) to classify microbial constituents and compare membership between samples. The following quality check parameters were used: minimum quality score of 27, no primer mismatch, read length of 300 to 400 bp, maximum 3 ambiguous bases and 6 bases in homopolymer runs. A total of 759,182 sequences were used to construct operational taxonomic units (OTUs) consisting of sequences with 97% sequence identity. Chimeric sequences detected with ChimeraSlayer (Haas et al., 2011) and OTUs comprising fewer than four reads were filtered from the dataset. As a result, 3563 OTUs with quality-filtered sequences were obtained. A representative sequence from each OTU was aligned to the Greengenes reference sequence database (gg_13_5) (DeSantis et al., 2006) with a confidence threshold value of 80% with the uclust (Edgar, 2010) consensus taxonomy assigner (pick_rep_set.py, assign_taxonomy.py). The bacterial composition of each sample was determined for each taxonomic rank by applying the summarize_taxa_through_plots.py function of QIIME to the OTU table with the assigned taxonomy dataset. For closest species, representative sequences for each OTU at information level 7 were identified by using the Basic Local Alignment Search Tool (BLAST) v2.2.29+ against the NCBI 16S Microbial database, with the threshold for sequence identity set at 97% and the *e*-value at 1e-4 (Supplementary Table S12). The raw sequence files supporting the findings of this article are available in the NCBI Sequence Read Archive under the BioProject ID PRJNA350740 (Biosamples SAMN06052019 to SAMN06052163). All data supporting the results of our study are included within this article and the Supplementary Materials.

### Principal Component Analysis

The bacterial family diversity of each breast milk sample was analyzed on the basis of family relative abundance by using principal component analysis (PCA). The R package (ggbiplot) was used to generate PCA plots by using the first two principal components according to group.

### Clustering Analysis

Clustering was performed in the R environment^1^ according to the enterotyping tutorial provided by the European Molecular Biology Laboratory^2^. We calculated the probability distribution distance metric related to Jensen–Shannon divergence, and we used a principal coordinate analysis (PCoA) with the partitioning around medoids (PAM) clustering algorithm to cluster the abundance profiles of samples at the family level. We used the Calinski–Harabasz index (Caliñski and Harabasz, 2007) to determine the optimum number of clusters in our dataset. The clusters obtained were validated by using the prediction strength (Tibshirani and Walther, 2005) and the silhouette width index (Rousseeuw, 1987).

### Microbiome Network Construction

To construct a co-occurrence network of the predominant breast milk microbiota of different clusters, we performed a bivariate correlation analysis for the 15 most abundant genera by using Spearman’s correlation coefficient in R, with a *P*-value of 0.05. The network was generated using Cytoscape (version 3.2.1) and visualized using a circular layout in which the nodes represented bacterial genera and the edges represented the strength of the positive (green) or negative (red) correlation between the genera. Node size indicated the abundance of each bacterial genus in our dataset, and the widths of the edges reflected the Spearman’s correlation values.

### Alpha- and Beta-Diversities of Bacterial Communities

Alpha diversity [as measured by the number of species observed, the phylogenetic diversity (PD) whole-tree, and Chao1 indices] and Beta diversity were estimated with QIIME. Individual OTU composition data were rarefied by using 1000 reads per sample in 10 random iterations. The average values of the number of observed OTUs, the PD whole tree, and the Chao1 indices over 10 iterations were calculated for each rarefied OTU composition. Beta diversity analysis was performed by using both phylogenetic and non-phylogenetic distance matrices. Phylogenic relationships and OTU abundances were simulated by UniFrac analysis (Lozupone and Knight, 2005), and compositional dissimilarity according to OTU abundance was quantified on the basis of the Bray–Curtis metric. Two-dimensional PCoA plots were generated by using the make_2d_plots script bundled with QIIME. The QIIME script compare_categories using an ANalysis Of SIMilarity (ANOSIM) (Clarke, 1993) test was used to consider whether there were significant differences in sample groupings using distance matrices.

### Statistical Analysis

All statistical analyses were performed with R software. The associations between the three clusters found and the seven geographical regions were analyzed by using Pearson’s Chi-squared test. The enrichment of clusters in each region was assessed by using a hyper-geometric distribution test (Ma et al., 2015). The Kruskal–Wallis test was used to identify significant differences in the abundance of bacterial compositions between the seven geographical regions and the three clusters. All *P*-values were corrected by the false discovery rate (FDR) using the Benjamini–Hochberg method (Benjamini and Hochberg, 1995) for multiple comparisons. The Mann–Whitney *U*-test was calculated for comparison of the two delivery mode groups (vaginal and cesarean). Fisher’s exact test was applied to compare the prevalence of microbial detection between the three clusters.

## Results

### Subjects

A total of 133 healthy mothers (21–42 years old; mean 28.5 ± 4.6) were recruited into the study. The number of volunteers recruited from each region was as follows: Central China, *n* = 24; East China, *n* = 34; North China, *n* = 11; Northeast China, *n* = 17; South China, *n* = 7; Southwest China, *n* = 9; Taiwan, *n* = 31. The raw physical characteristics and the lactation stage (months postpartum) when the milk samples were collected are shown in Supplementary Table S1. There were no significant differences in age or BMI among the regions, clusters, and delivery mode groups, except in the case of the mothers from Taiwan. The average age of mothers from Taiwan was significantly higher than those of the mothers from five of the other regions (*P* < 0.001 vs. Central, East, Northeast, and Southwest; *P* < 0.05 vs. North China). The BMI of mothers from Taiwan was significantly lower (*P* < 0.05) than that of mothers from North China. The average stage of lactation of all of the mothers was 6.1 ± 4.0 months (mean ± SD) after delivery, and this parameter did not differ significantly among the regions, clusters, or groups. The 131 milk samples excluding two samples which had no data of their body weight were stratified by maternal BMI into three groups: (i) <18.5 (*n* = 12), (ii) 18.5–25.0 (*n* = 87), and (iii) >25.0 (*n* = 32). There were no significant differences in the abundances of the 17 most predominant bacterial families among the samples collected from these three different BMI groups (data not shown).

### Global Differences in Breast Milk Bacterial Communities among Women Living in Taiwan and Mainland China

We obtained a total of 746,312 high-quality filtered reads, or 5611.4 ± 4141.5 reads per participant. The reads were clustered into 3563 OTUs, and representative sequences were used in the taxonomic analysis. The OTUs were classified into known taxa (16 phyla, 40 classes, 71 orders, 134 families, 245 genera, and 98 species) and unclassified groups. Taxonomic and phylogenetic information on the OTUs is provided in Supplementary Table S2.

**FIGURE 1:**
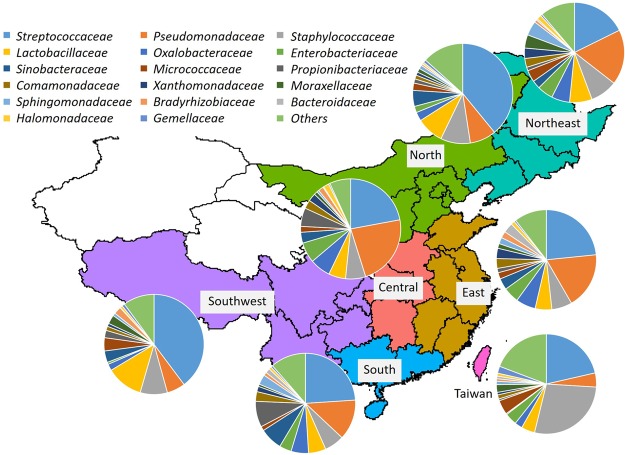
Bacterial community composition in breast milk samples from mothers living in Taiwan and mainland China. Bacterial families were identified by 454-pyrotag sequencing of 16S rRNA genes. Each pie chart shows the mean composition of bacterial families in each region.

The geographical distribution of bacterial families in breast milk is illustrated in **Figure 1**. The relative abundances of the bacterial families in the seven regions are shown in Supplementary Table S3. The 10 predominant families detected were *Streptococcaceae* (mean relative abundance: 24.4%), *Pseudo-monadaceae* (14.0%), *Staphylococcaceae* (12.2%), *Lactobacillaceae* (6.2%), *Oxalobacteraceae* (4.8%), *Enterobacteriaceae* (4.5%), *Sinobacteriaceae* (3.1%), *Micrococcaceae* (3.0%), *Propionibac-teriaceae* (2.4%), and *Comamonadaceae* (2.4%). The abundance of *Pseudomonadaceae* (4.6%) was significantly lower in samples from Taiwan than in those from East (*P* < 0.01), Central (*P* < 0.001), and Northeast (*P* < 0.05) China, whereas the abundance of *Staphylococcaceae* (27.9%) was significantly higher in samples from Taiwan than in those from East China (*P* < 0.01) and Central China (*P* < 0.05). The abundance of *Pseudomonadaceae* (23.1%) was significantly higher (*P* < 0.05) in samples from Central China than in those from North and Southwest China.

### Identification of Three Clusters

**FIGURE 2:**
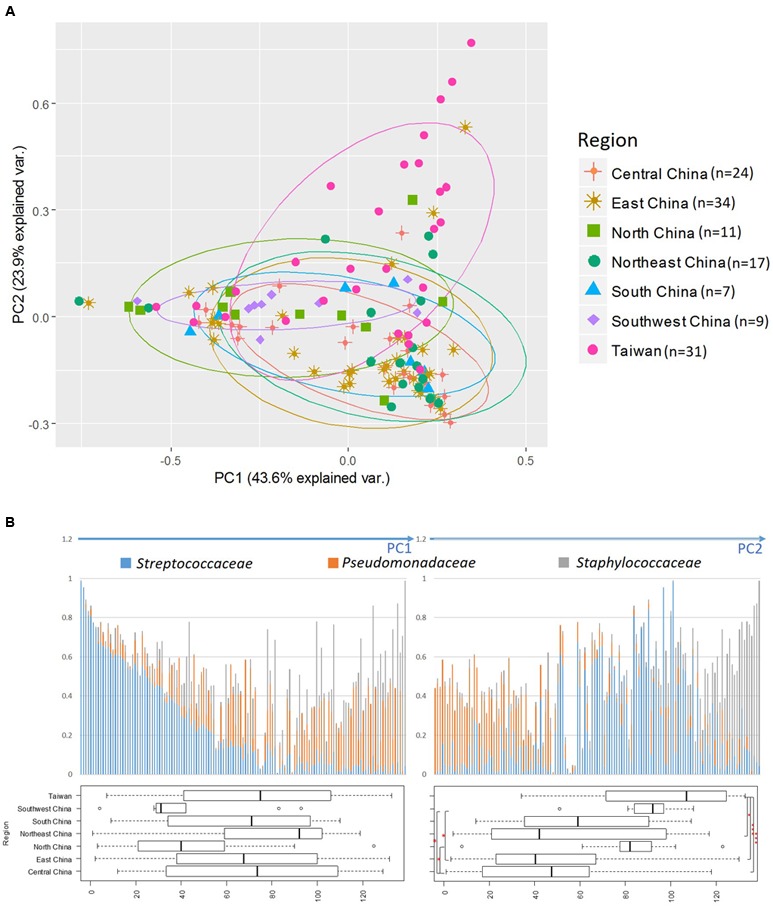
Principal component analysis (PCA) of bacterial abundance. **(A)** Principal component analysis (PCA) performed by using relative abundance data on all bacterial families. The three most abundant bacterial families are indicated by arrows and family names. **(B)** Distribution of the three predominant families in the 133 breast milk samples. The bacterial-composition data were sorted according to the PC1 score (left) and PC2 score (right). Variance among participants from each region is shown as a boxplot showing the smallest and largest values, 25% and 75% quartiles, the median, and outliers. ^∗^*P* < 0.05; ^∗∗∗^*P* < 0.001.

By using PCA, we decomposed the data on bacterial families into two factors that explained 67.5% of the variance (**Figure 2A**). Principal component 1 (PC1) was heavily loaded with *Streptococcaceae*, whereas principal component 2 (PC2) was heavily loaded with *Staphylococcaceae* and *Pseudomonadaceae*. Samples from Taiwan were strongly influenced by *Staphylococcaceae*, whereas those from North and Southwest China were influenced by *Streptococcaceae*. The frequencies of the three predominant bacterial families in all samples are shown in **Figure 2B**. The PC1-ordered graph shows a PC1-negative region carrying a distinctive core of *Streptococcaceae* (left panel). The PC2-ordered graph shows two types of microbiota: across the PC2-positive regions carrying a distinctive core of *Staphylococcaceae*, and across the PC2-negative regions carrying a distinctive core of *Pseudomonadaceae* (right panel). The box plots in **Figure 2B** show that samples from Taiwan were distributed in the PC2-positive region (*P* < 0.001 vs. Central and East; *P* < 0.05 vs. Northeast China). Samples from Southwest China were also distributed in the PC2-positive region (*P* < 0.05 vs. East and Central China).

**FIGURE 3:**
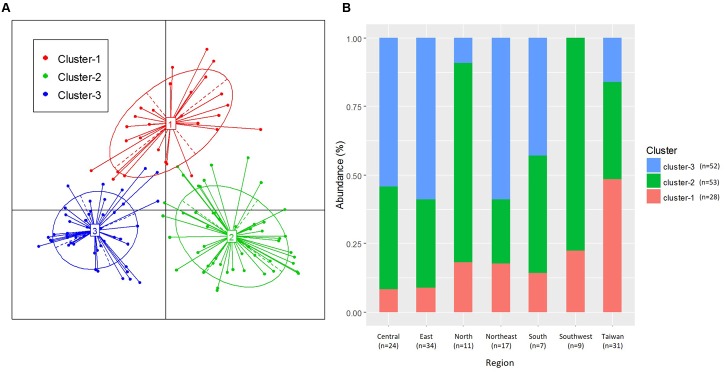
Clustering of breast milk samples from 133 mothers, based on bacterial family composition data. **(A)** Jensen–Shannon divergence and Partitioning Around Medoids (PAM) clustering were used. The optimal number of clusters was chosen by maximizing the Calinski–Harabasz index and was validated on the basis of the prediction strength and average silhouette width indices. **(B)** Ratio of the three clusters in each region.

We examined the clustering of all samples by using PCoA with the PAM clustering algorithm (Supplementary Table S1); the optimum cluster number at the family level was three, with a silhouette width of 0.22 and a prediction strength of 0.71 (**Figure 3A** and Supplementary Table S4). This clustering into three groups was highly correlated with the results of the PCA influenced by the three major bacterial families, namely *Staphylococcaceae*, *Streptococcaceae*, and *Pseudomonadaceae*.

**FIGURE 4:**
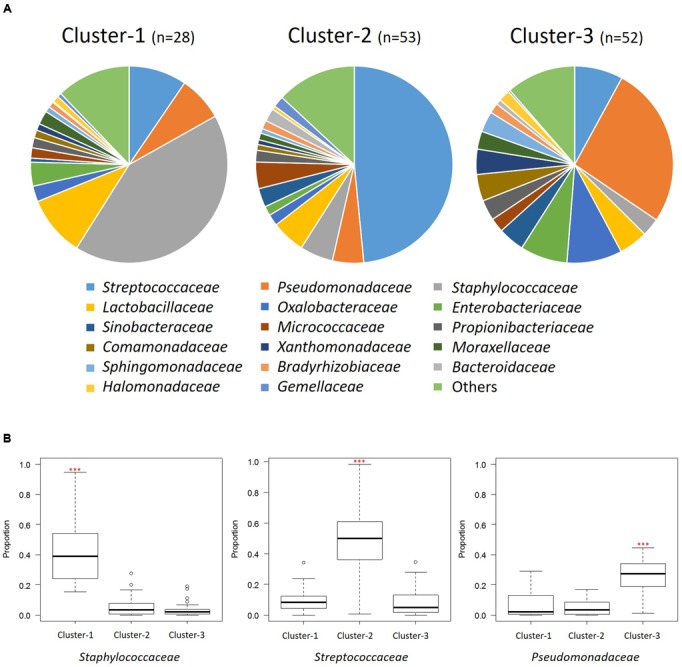
Bacterial community composition of the three clusters at the family level. **(A)** Pie charts show the mean relative ratios of the bacterial families in each cluster. **(B)** The relative abundances of the three predominant families in each cluster (^∗∗∗^*P* < 0.001 in the Kruskal-Wallis test with FDR correction). Box plot showing the smallest and largest values, 25% and 75% quartiles, the median, and outliers.

The distribution of the three clusters in each region is shown in **Figure 3B**. We confirmed a significant difference (*P* < 0.001) in the associations between the three clusters and seven geographical regions (Supplementary Table S5A). We assessed the enrichment of clusters in each region and confirmed the characteristic enrichment of each cluster: Cluster 1 was predominant in Taiwan (*P* < 0.001), Cluster 2 in North and Southwest China (*P* < 0.01), and Cluster 3 in Central and Northeast China (*P* < 0.05) and East China (*P* < 0.01) (Supplementary Table S5). Cluster 1 was driven by *Staphylococcaceae* (*P* < 0.001; abundance: 42.1%), Cluster 2 by *Streptococcaceae* (*P* < 0.001; 48.5%), and Cluster 3 by *Pseudomonadaceae* (*P* < 0.001; 26.5%). The abundances of *Staphylococcaceae* in Cluster 1, *Streptococcaceae* in Cluster 2, and *Pseudomonadaceae*, *Oxalobacteraceae*, *Enterobacteriaceae*, *Comamonadaceae*, *Xanthomonadaceae*, *Sphingomonadaceae*, and *Halomonadaceae* in Cluster 3 were significantly higher than those in other clusters (**Figure 4** and Supplementary Table S6). The 15 genera with the highest relative abundances in all 133 samples and in each of the three clusters were regarded as the predominant breast milk bacteria in the respective sample sets. In all samples, the predominant genera were *Streptococcus*, *Pseudomonas*, *Staphylococcus*, *Lactobacillus*, *Propionibacterium*, *Herbaspirillum*, *Rothia*, *Stenotrophomonas*, *Acinetobacter*, *Bacteroides*, *Halomonas*, *Veillonella*, *Sphingomonas*, *Delftia*, and *Corynebacterium*. Among the 17 predominant genera in each cluster, the abundances of *Staphylococcus* (42.1%) in Cluster 1, *Streptococcus* (48.4%) in Cluster 2, and *Pseudomonas* (26.3%) in Cluster 3 were significantly higher (*P* < 0.001) than those in the other clusters (Supplementary Table S7).

### Network of Co-occurring Predominant Bacterial Genera

**FIGURE 5:**
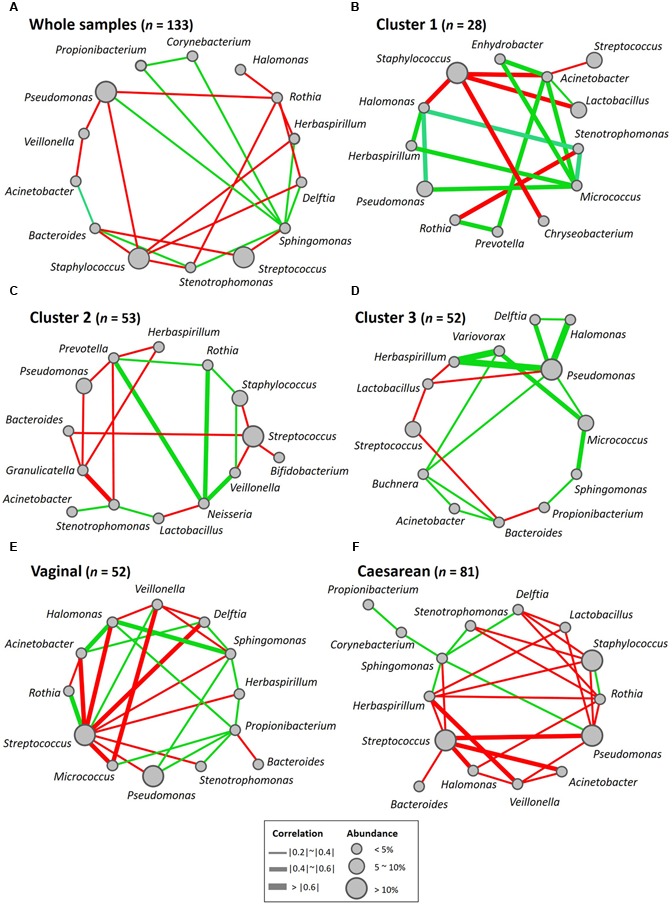
Bacterial networks of the breast milk microbiome. Networks of co-occurring genera were constructed in Cytoscape by using correlation data on the core milk bacteria in **(A)** whole samples, **(B)** Cluster 1, **(C)** Cluster 2, **(D)** Cluster 3, **(E)** women who had delivered vaginally, and **(F)** women who had delivered by cesarean section. Nodes represent bacterial genera, and edges represent the strength of positive (green) or negative (red) correlations between the genera.

To further explore bacterial interactions within the milk microbiota, we constructed a milk bacterial community network. In the microbiome network constructed from the 133 samples, there were significant positive or negative correlations (*P* < 0.05 and |r| = 0.21 to 0.33) among the abundances of 14 of the 15 predominant genera (excluding *Lactobacillus*). The abundance of at least one of these genera was significantly correlated (*P* < 0.05 and |r| > 0.21) with that of at least one of 13 other bacterial genera, among which *Rothia*, *Staphylococcus*, and *Streptococcus* variously had negative correlations with *Bacteroides*, *Delftia*, *Herbaspirillum*, *Pseudomonas*, and *Sphingomonas* (**Figure 5A** and Supplementary Table S8A). Likewise, there were significant positive or negative correlations (*P* < 0.05 and |r| = 0.39 to 0.59) among 13 genera (excluding *Corynebacterium* and *Propionibacterium*) in the Cluster 1 samples, among which *Staphylococcus* had negative correlations with *Acinetobacter*, *Chryseobacterium*, and *Halomonas* (**Figure 5B** and Supplementary Table S8B). There were significant positive or negative correlations (*P* < 0.05 and |r| = 0.28 to 0.50) among 14 genera (excluding *Propionibacterium*) in the Cluster 2 samples, among which *Streptococcus* had negative correlations with *Bacteroides*, *Bifidobacterium*, and *Staphylococcus* (**Figure 5C** and Supplementary Table S8C). There were significant positive or negative correlations (*P* < 0.05 and |r| = 0.27 to 0.82) among 13 genera (excluding *Staphylococcus* and *Stenotrophomonas*) in the Cluster 3 samples, among which *Pseudomonas* had positive correlations with *Buchnera*, *Delftia*, *Halomonas*, *Herbaspirillum*, and *Micrococcus* (**Figure 5D** and Supplementary Table S8D), respectively. On the whole, the Gram-positive *Staphylococcus*, *Streptococcus*, and *Rothia* showed negative correlations with the Gram-negative *Acinetobacter*, *Bacteroides*, *Halomonas*, *Herbaspirillum*, and *Pseudomonas*.

### Alpha- and Beta-Diversities of Bacterial Communities

**FIGURE 6:**
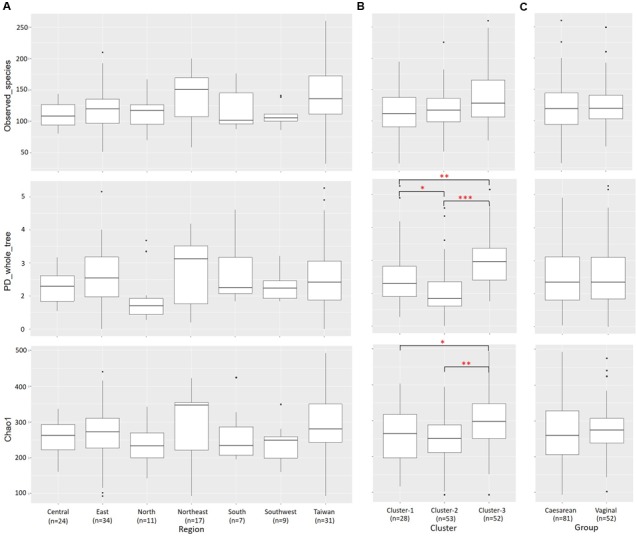
Alpha diversities of bacterial communities in individual samples from each region and cluster. Individual OTU-composition data were rarified by using 1000 reads per sample in 10 iterations. Number of observed OTUs, PD whole-tree values, and Chao1 indices were calculated for each rarefied OTU composition and averaged within the 10 iterations. The covariances of these indices were computed for each **(A)** region, **(B)** cluster and **(C)** delivery mode group and graphed as box plots. ^∗^*P* < 0.05; ^∗∗^*P* < 0.01; ^∗∗∗^*P* < 0.001.

Alpha diversity (within-community diversity) was compared among regions and clusters (**Figure 6** and Supplementary Table S1). The microbiota in Cluster 3 samples showed significantly higher PD whole-tree and Chao1 diversity indices than those in Cluster 1 samples (*P* < 0.05) and Cluster 2 samples (*P* < 0.001) (**Figure 6B**).

**FIGURE 7:**
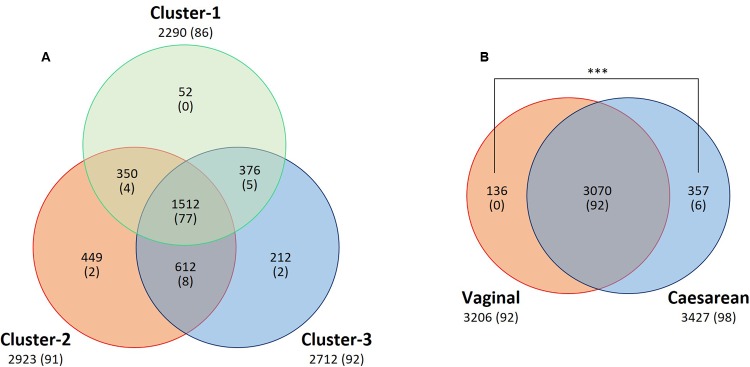
Venn diagram of core OTU (or known bacterial species) sharing among **(A)** clusters or **(B)** delivery mode groups. The numbers of shared and unique OTUs (or known species) are shown. Numbers of known species are shown in parentheses. A significantly greater (*P* < 0.001) number of unique OTUs was found in the breast milk from mothers who had undergone Caesarian section than from those who had delivered vaginally. ^∗∗∗^*P* < 0.001.

A total of 2290 OTUs (including 86 species) were ascribed to Cluster 1; we ascribed 2923 OTUs (including 91 species) to Cluster 2 and 2712 OTUs (including 92 species) to Cluster 3, and 1512 OTUs (including 77 species) were common to the three clusters. In pairwise comparisons between clusters, the numbers of common OTUs and species were 350 and 4 (Clusters 1 and 2), 612 and 8 (Clusters 2 and 3), and 376 and 5 (Clusters 1 and 3), respectively (**Figure 7A**).

**FIGURE 8:**
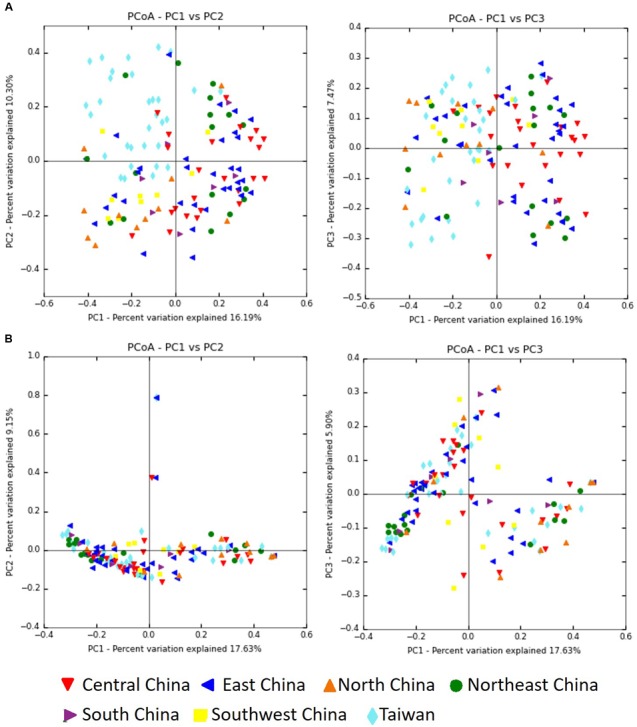
Principal coordinate analysis (PCoA) profiles of microbial diversity across all samples. The percentages of variation explained by PC1, PC2, and PC3 are indicated on the axes. **(A)** PCoA plots based on Bray–Curtis dissimilarity metric. **(B)** PCoA plots based on unweighted UniFrac metric.

The Bray–Curtis dissimilarity metric was used to examine both the differences and the similarities in the microbial community, with consideration of both the occurrence and the abundance of OTUs. Comparison of the bacterial communities of the seven regions on the basis of the Bray–Curtis dissimilarity metric showed that these communities differed in both composition and abundance of OTUs (*P* = 0.001). Differences between the communities of the seven regions were also observed in PCoA plots created by using the Bray–Curtis distance matrices. **Figure 8A** shows clustering of the samples from Taiwan, whereas the samples from mainland China exhibited a more dispersed distribution. In contrast, clustering of samples based on a weighted (quantitative) and unweighted (qualitative) UniFrac analysis showed no significant differences for sample groupings (*P* = 0.818, *P* = 0.249, respectively). In both the weighted (data not shown) and unweighted UniFrac distances, there was no separation into the clusters observed in the PCoA plots (**Figure 8B**).

### Distribution of Lactobacilli and Bifidobacteria

A total of four *Lactobacillus* species (*Lactobacillus paracasei*, *L. reuteri*, *L. rhamnosus*, and *L. vaginalis*) and five species of *Bifidobacterium* (*Bifidobacterium adolescentis*, *B. dentium*, *B. longum*, *B. longum* subsp. *infantis*, and *B. stercoris*) were detected. *L. paracasei* (prevalence: 78.2%, abundance: 3.2%) was the most abundant species among the 98 known species detected in whole samples (*n* = 133). *B. longum* (62.4%, 0.3%) was the predominant bifidobacterial species (Supplementary Tables S9, S10). The abundance of *L. rhamnosus* in Cluster 1 samples was significantly higher than those in Cluster 2.

### Impact of Lactation Stage and BMI on Relative Abundances of Bacterial Components in Breast Milk

There were no significant differences in lactation stage at which samples were taken among the seven geographical regions, three clusters, and two delivery mode groups. To analyze the impact of lactation stage on breast milk microbiota, the 133 milk samples were stratified by lactation stage into three groups: (i) less than 3 months (*n* = 28), (ii) 3–6 months (*n* = 50), and (iii) more than 6 months (*n* = 55). There were no significant differences in the abundances of the 17 most predominant bacterial families among samples collected at the three different lactation stages (Supplementary Table S11).

### Effect of Delivery Mode on Relative Abundances of Bacterial Components in Breast Milk

To examine whether the delivery mode (vaginal vs. cesarean) affected the breast milk microbiota, we compared the relative abundances of different bacterial families or genera in these two groups. Milk from mothers who had undergone caesarian section (C-section group; *n* = 81) had a significantly greater abundance of *Lactobacillus* (*P* = 0.033) than milk from mothers who had delivered vaginally (vaginal group; *n* = 52), but there were no significant differences in the relative abundances of bacterial families in milk between the two groups (Supplementary Tables S7, S11). A total of 3070 OTUs (including 92 known species) were observed as common OTUs or species to the two groups. Group-specific OTUs or species were also detected: 136 OTUs in the vaginally delivered group, and 357 OTUs (including six species) in the C-section group. The number of group-specific unclassified OTUs in the C-section group was significantly higher (*P* < 0.001; Fisher’s exact test) than that in the vaginal group (**Figure 7B**). However, there were no significant differences in the alpha-diversity indices between the two groups (**Figure 6C**). Microbiome network analysis of interactions within the 15 predominant bacterial genera in the samples revealed significant positive or negative correlations (*P* < 0.05 and |r| = 0.29 to 0.51) among the abundances of 13 of the 15 predominant genera (excluding *Lactobacillus* and *Staphylococcus*) in the vaginal delivery group. Likewise, there were significant positive or negative correlations (*P* < 0.05 and |r| = 0.24 to 0.42) among the 15 predominant genera in the C-section group (**Figures 5E,F** and Supplementary Table S8).

## Discussion

### Microbial Profile

By using 454 pyrosequencing of 16S rRNA genes, we identified 3563 OTUs in 133 breast milk samples collected from healthy mothers living in Taiwan and in six regions of mainland China. Although our study was limited by low sequence depth, to our knowledge it is the first to have comprehensively investigated the microbial diversity in breast milk from mothers living across different geographical regions of East Asia.

The 10 most abundant bacterial families detected in our study were *Streptococcaceae*, *Pseudomonadaceae*, *Staphylococcaceae*, *Lactobacillaceae*, *Oxalobacteraceae*, *Enterobacteriaceae*, *Sinobacteriaceae*, *Propionibacteriaceae*, *Micrococcaceae*, and *Comamonadaceae*; these results are generally consistent with those of previous studies (Martín et al., 2007b; Hunt et al., 2011; Jeurink et al., 2012; Ward et al., 2013; Jiménez et al., 2015; Urbaniak et al., 2016). Kumar et al. (2016) reported that geographical location directly affected the microbiota and fatty acids in breast milk samples from women in China, Finland, South Africa, and Spain. We observed significant differences in microbial composition among samples collected from various regions. The microbiota from all samples was classified into three clusters, driven by *Staphylococcaceae* (abundance in Cluster 1: 42.1%), *Streptococcaceae* (Cluster 2: 48.5%), or *Pseudomonadaceae* (Cluster 3: 26.5%). In Cluster 1, *Staphylococcaceae* and *Lactobacillaceae* made up more than half (52.0%) of the total microbiota.

By using the microbial network analysis method, Ma et al. (2015) found interactions within the milk microbiota: the abundances of the Gram-positive *Staphylococcus* and *Corynebacterium* were negatively correlated with those of the Gram-negative *Bradyrhizobium*, *Pseudomonas*, and *Sphingomonas*, whereas that of the Gram-positive *Propionibacterium* was positively correlated with those of the Gram-negative *Pseudomonas*, *Ralstonia*, *Serratia*, and *Sphingomonas*. In our network analysis, we found that the abundances of the Gram-positive *Staphylococcus*, *Streptococcus*, and *Rothia* were variously negatively correlated with those of the Gram-negative *Acinetobacter*, *Bacteroides*, *Halomonas*, *Herbaspirillum*, and *Pseudomonas*.

A wide variety of skin- and enteric-associated Gram-negative bacteria observed in breast milk samples are potential opportunistic pathogens; however, our results suggest that the growth of these is suppressed by Gram-positive bacteria such as *Staphylococcus* and *Rothia*. In particular, *Staphylococcus* (predominant in Cluster 1) may be a key factor controlling microbial interactions.

Sakwinska et al. (2016) analyzed the microbiota of breast milk from mothers living in China and found differences between samples collected by using a standard protocol with and without aseptic cleansing. The microbiota of breast milk was dominated by streptococci and staphylococci; however, *Acinetobacter* sp. was predominant at high abundance (30%) when a standard collection protocol without aseptic cleansing of breast and rejection of the foremilk was used. The milk samples that we analyzed were collected over a large range of lactation stages (0.1–21.7 months; mean 6.1 ± 4.0 months after delivery), and five species of *Acinetobacter* were detected, but the overall abundance of *Acinetobacter* was very low (1.5%) (Supplementary Tables S6, S8). Hence, the abundance of *Acinetobacter* is negatively correlated not only with the abundance of the predominant *Staphylococcus*, but also with the use of an aseptic protocol for milk collection.

### Impact of Lactation Stage on Microbial Profiles in Breast Milk

Previous studies have revealed that the bacterial diversity in breast milk changes with lactation stage, from colostrum to transitional milk to mature milk. Boix-Amorós et al. (2016) confirmed that the bacterial microbiota in the milk of Spanish mothers changed in different lactation stages: the most common genus in colostrum was *Staphylococcus*, followed by *Acinetobacter*; *Pseudomonas*; and *Streptococcus* in transitional milk; and *Acinetobacter* in mature milk samples. However, we found no significant differences in the abundances of the 17 most abundant bacterial families among samples collected in the three different lactation stages (less than 3 months, 3–6 months, and more than 6 months) (Supplementary Table S11).

### Effect of Delivery Mode on Microbial Profiles in Breast Milk

Previous studies have investigated whether the delivery mode (vaginal delivery vs. C-section) affects microbiota profiles in breast milk. Higher abundances of *Leuconostocaceae* (predominant genus: *Leuconostoc*) and lower abundances of *Carnobacteriaceae* (*Carnobacterium*) and *Moraxellaceae* (*Acinetobacter*) have been observed in samples from mothers who have delivered vaginally than in samples from women who have delivered by C-section (Cabrera-Rubio et al., 2012). Greater bacterial diversity and richness (in particular, more *Bifidobacterium* species) have been found in samples from mothers who have delivered by C-section and fewer *Staphylococcus* species in samples from mothers who have delivered vaginally (Cabrera-Rubio et al., 2016). Kumar et al. (2016) also analyzed the microbiota in milk samples from women living in Spain, Finland, China, and South Africa who had delivered vaginally or by C-section; they observed significant differences in milk microbial profiles between the two delivery mode groups. In contrast, Sakwinska et al. (2016) and Urbaniak et al. (2016) observed no differences in microbial profiles between samples from mothers who had delivered vaginally and those who had delivered by C-section. However, these results were based on small-scale analyses. In our study, although we found no significant differences in the predominant microbial components in milk, the abundance of *Lactobacillus* was significantly higher (*P* < 0.05) after C-section than after vaginal delivery. Moreover, we observed a significantly greater (*P* < 0.001) number of unique OTUs in the breast milk microbiota of mothers who delivered by C-section than in the milk microbiota of mothers who delivered vaginally; however, no significant differences were found in the alpha diversity comparisons between these two groups (**Figures 6**, **7B**).

### Study Limitations

As described above, our study is the first comprehensive investigation of the breast milk microbiota of 133 mothers living in Taiwan and in six regions of mainland China. However, this study had limitations with respect to the sampling conditions (Supplementary Table S1). The numbers of milk samples collected among the seven regions were inconsistent (ranged from seven to 34 samples). Also, breast milks were obtained from mothers in a large range of lactation stages (mean: 6.0 ± 4.0 months; range: 0.1–21.7 months; median: 5.4) among the 133 samples. In additional, the milk samples had low sequencing depth (mean 5,611.4 ± 4,141.5 reads per sample; range: 1,008–19,345 reads; median: 4,287 reads). Lastly, we could not obtain the maternal information (in respect to dietary habits or antibiotic use during pregnancy) which could be relevant to the breast milk microbial profiles.

We consider that the milk samples collected evenly from each region at early lactation stage (less than 3 months after delivery) with sufficient high-quality sequences would provide more detailed microbial features of breast milk from East Asian women who delivered vaginally or by C-section.

Traditionally, human milk is considered to be colonized by bacteria from the mother’s gut and skin or the infant’s mouth (Makino et al., 2013; Rodríguez, 2014). Therefore, the mode of delivery has been discussed as a key determinant of the types of bacteria involved in early colonization of the infant gut. Martin et al. (2016) reported that infants born by C-section had delayed colonization by several bacterial groups or species, such as *Bacteroides* spp. and *Bifidobacterium* spp., whereas vaginal delivery in combination with breastfeeding favors colonization by *B. bifidum* and the *L. gasseri* subgroup (taxonomically defined as the *L. delbrueckii* group comprising *L. acidophilus*, *L. crispatus*, *L. delbrueckii*, *L. gasseri*, and *L. johnsonii*). Extensive studies of the fecal microbiota of Asian people have revealed that diet, ethnicity, and geography are key determinants of diversity in the fecal microbial composition (Nakayama et al., 2015, 2017; Zhang et al., 2015). Furthermore, gut microbiota composition is strongly associated with obesity (Turnbaugh et al., 2006, 2009; Boulangé et al., 2016). Here, we observed no significant effects of maternal age or BMI, or lactation stage, on the milk microbiota. However, it is important to consider not only these maternal factors (along with dietary habits or antibiotic use) but also the mothers’ fecal microbiota as key factors shaping the breast milk microbiota.

The contradictions between our results and these previous results may have been due to differences in the analytical methods used. The factors that influence the breast milk microbiota are still largely unknown, and further investigations are needed to shed light on microbial dynamics. Studies of the relationship between the maternal fecal microbiota and breast milk microbiota would contribute to a better understanding of the benefits of breastfeeding for infants.

## Conclusion

We used 16S rRNA pyrosequencing to characterize the breast milk microbiota in 133 samples from mothers living in Taiwan and six regions of mainland China. Geographical differences were found in the microbial profiles. The predominant bacterial families *Streptococcaceae*, *Staphylococcaceae*, and *Pseudomonadaceae* were key components of the three clusters found. A significantly greater number of unique OTUs was found in the breast milk from mothers who had undergone Caesarian section than from those who had delivered vaginally.

## Author Contributions

S-WL conducted most of the experiments, analyzed and interpreted the data, and wrote the manuscript. KW designed and oversaw the study, analyzed and interpreted the data, wrote the manuscript, and is a corresponding author. C-CH collected breast milk samples and questionnaire data. S-HC collected breast milk samples and questionnaire data, extracted breast milk DNA, and participated in data analysis. Z-HY, Y-JL, C-CC, and Y-MC collected breast milk samples. H-CH and C-HC oversaw the study and reviewed the manuscript. Y-CT supervised the study, reviewed the manuscript, and is a corresponding author. All authors read and approved the final manuscript.

## Conflict of Interest Statement

The authors declare that the research was conducted in the absence of any commercial or financial relationships that could be construed as a potential conflict of interest.

## Footnotes

1 http://www.r-project.org/

2 http://enterotype.embl.de/enterotypes.html
