# Supplementary material for: Bacterial Composition and Diversity in Breast Milk Samples from Mothers Living in Taiwan and Mainland China
Source: Front Microbiol. 2017 May 30;8:965. doi: 10.3389/fmicb.2017.00965 (PMC5447776; doi:10.3389/fmicb.2017.00965)
Supplement: Supplementary file 1 [file Data_Sheet_1.DOCX]

Supplementary Material

**Bacterial composition and diversity in breast milk samples from mothers living in Taiwan and mainland China**

Shiao-Wen Li^1, 2†^, Koichi Watanabe^3, 4, 5^*^†^, Chih-Chieh Hsu^5^, Shiou-Huei Chao^6^, Zheng-Hua Yang^7^, Yan-Jun Lin^7^, Chun-Chiang Chen^7^, Yong-Mei Cao^7^, Hsuan-Cheng Huang^1^, Chuan-Hsiung Chang^1^, Ying-Chieh Tsai^6^*

*** Correspondence:**

Koichi Watanabe, e-mail: koichi_wtnb@yahoo.co.jp, Tel: 886-2-3366-4169; Fax: 886-2-2732-4070

Ying-Chieh Tsai, e-mail: tsaiyc@ym.edu.tw, Tel: 886-2-2826-7125; Fax: 886-2-2826-4843

**SUPPLEMENTARY MATERIAL**

**Supplementary: Table S1.** Subject characteristics and the results of pyrosequencing. **Table S2.** Operational taxonomic units identified in breast milk samples from 133 healthy mothers living in Taiwan and Mainland China. **Table S3.** Relative abundances of 17 predominant bacterial families in the 133 breast milk samples (by region). **Table S4.** Cluster analysis at each taxonomic rank. **Table S5.** Distribution of three clusters in each geographical region. **Table S6.** Relative abundances of 17 predominant bacterial families in the 133 breast milk samples (by cluster). **Table S7.** Relative abundances (%) of 17 predominant bacterial genera in the 133 breast milk samples. **Table S8.** Correlations among 15 predominant bacterial genera in the 133 breast milk samples. **Table S9.** Relative abundances and prevalences of bacterial species in the 133 breast milk samples. **Table S10.** Relative abundances and prevalences of *Lactobacillus* and *Bifidobacterium* species in the 133 breast milk samples. **Table S11.** Relative abundances of 17 predominant bacterial families in breast milk samples from mothers between who had delivered vaginally or by Caesarean section and between three different lactation stages. **Table S12.** Quantitative Insights Into Microbial Ecology (QIIME) bioinformatics pipeline scripts used in the present study.
